# To Start or Not to Start—A Matter of Genetics in Swedish Warmblood Horses?

**DOI:** 10.1111/jbg.70005

**Published:** 2025-07-25

**Authors:** Åsa Gelinder Viklund, Ylva Blom, Susanne Eriksson

**Affiliations:** ^1^ Department of Animal Biosciences Swedish University of Agricultural Sciences Uppsala Sweden

**Keywords:** dressage, jumping, sport horses, start status, young horse test

## Abstract

The breeding goal of the Swedish Warmblood horse (SWB) is to produce internationally competitive horses in dressage and show jumping. In the current genetic evaluation, breeding values are estimated in multiple‐trait animal models where competition performance is the target trait and results from two different young horse tests serve as indicator traits. However, preselection of horses, both for young horse tests and for competitions, is not considered in the current evaluation. The overall aim of this study was to analyse the all‐or‐none trait start status, in competition and in young horse tests, for possible use in the genetic evaluation for SWB. All starts in young horse tests have been recorded since long (1973), whereas start status in competition is known from the year 2007 and onwards. Therefore, the studied population was restricted to SWB horses born between 2003 and 2018 that had the possibility to compete during the period from 2007 until 2022. Horses were categorised into four disciplines according to their sire's and grandsire's discipline categories, and only horses in the two major categories, dressage and jumping, were included in this study. In total, 23,125 jumping horses and 14,470 dressage horses were studied separately. Information on discipline‐specific start status in show jumping or dressage competitions, young horse test (YHT) and riding horse test (RHT) was available as well as lifetime accumulated competition points, assessed gaits and jumping traits from YHT and RHT. Out of the jumping horses, 31% had participated in YHT, 10% in RHT and 56% in show jumping competition. For dressage horses, the participation rates were 35% for YHT, 11% for RHT and 34% for dressage competition. The genetic analyses were performed with threshold and linear animal models. Horses that had participated in YHT or RHT had competed to a larger extent and had a higher mean of competition points than horses that had not participated in YHT or RHT. The heritability for start status in competition was estimated using a threshold model at 0.48 for show jumping and 0.39 for dressage. Using linear models, the heritability for start status in show jumping was estimated to be 0.30 on the observable 0/1‐scale and 0.47 when transformed to the underlying continuous scale. For start status in dressage, the corresponding heritability estimates were 0.20 and 0.34. Genetic correlations, estimated with linear models, were strong between start status in show jumping and jumping traits at YHT and RHT (0.78–0.93) and moderate to strong between start status in dressage competition and gait traits at YHT and RHT (0.46–0.88). The genetic correlations between start status and accumulated lifetime points in competition were strong, 0.93 for show jumping and 0.86 for dressage. Using linear models, heritability estimates for start status in young horse tests ranged from 0.07 to 0.42 on the observable scale and from 0.11 to 0.71 after transformation to the underlying continuous scale. Inclusion of start status in the breeding value estimation of competition performance affected stallion ranking somewhat and increased the accuracies of the stallions' breeding values. We conclude that start status is a heritable trait that would be possible to include in the genetic evaluation of SWB horses.

## 1 Introduction

The breeding goal of the Swedish Warmblood horse (SWB) is to produce internationally competitive horses in show jumping and dressage (Swedish Warmblood Association 2021). Competition results have been included in the genetic evaluation since 2006 (Viklund et al. 2011). Breeding values are estimated in multiple‐trait animal models where competition performance is the target trait and results from two different young horse tests serve as indicator traits. Using all three information sources together implies an advantage because young horse tests include less selected data than competition data (Viklund, Braam, et al. 2010). However, there is still preselection of horses, both to young horse tests and to competitions, that is not considered in the current genetic evaluation. This preselection may cause biased breeding values unless it is considered in the genetic evaluation (Albertsdóttir et al. 2011; Árnason et al. 2012).

Inclusion of the all‐or‐none trait start status, defined as whether or not a horse participated in for example, breeding shows, young horse tests or competitions, in the genetic evaluation has been shown to reduce bias due to preselection, affect the ranking of stallions and increase accuracies of breeding values (Árnason 1999; Bugislaus et al. 2005; Albertsdóttir et al. 2012). Start status has been studied in several horse populations, for example in Swedish, German and French trotters (Árnason 1999; Bugislaus et al. 2005; Langlois and Vrijenhoek 2004), Icelandic horses (Albertsdóttir et al. 2011), and in Dutch and Belgian warmblood sport horses (Ducro 2010; Rovere et al. 2016; Janssens et al. 2007; Chapard et al. 2023). In these studies, heritability estimates for start status varied widely from low to high (ranged from 0.16 to 0.72) and the genetic correlations with performance or test traits varied between zero and strong (0 and 0.89).

In the current genetic evaluation of SWB, lifetime accumulated points is used as a measure of competition performance (Viklund, Braam, et al. 2010). Points are given to the 25% best horses in each competition, and more points are given for better placings and higher levels of competition. Competition data from the early 1960s are used in the genetic evaluation, and the recording of results has changed over time. Until 2006, only horses that were placed in a competition were recorded, meaning that horses that had started in competition but not been placed were missing from the data. Since 2007, all started horses are recorded, so started horses without a placing are included with zero points. One can argue that horses that have started in competition are probably on average better than horses that have not started at all, even if they were not placed.

The SWB population has become specialised into show jumping and dressage horses during the last decades (Bonow et al. 2023). In contrast to trotters, Icelandic horses and Belgian Warmblood jumping horses, all SWB horses are therefore not bred to compete in the same type of discipline. Even if there is no formal division between show jumping and dressage horses in the SWB, like in the Dutch Warmblood (KWPN) where foals are registered as either show jumping or dressage in the studbook since 2006 (Rovere et al. 2016), start status in the discipline the horse is not aimed for is less relevant than in the targeted discipline. Few (< 10%) SWB jumping horses are competing in dressage and vice versa (Bonow et al. 2023), and genetic correlations between traits related to dressage and jumping are low (Viklund, Braam, et al. 2010). SWB horses bred for show jumping and dressage have been shown to have significantly different mean scores for young horse test traits related to gaits as well as jumping (Bonow et al. 2024). Besides genetic differences between these groups of horses, it is reasonable to assume that the preselection for show or competition purposes is more rigorous with respect to traits relevant to the horses' intended discipline. In Icelandic horses, the preselection was shown to be strongest for the traits perceived as most valuable for the breed, such as conformation of neck, withers and shoulders, proportions, and gait traits (Albertsdóttir et al. 2011).

The overall aim of this study was to analyse the all‐or‐none trait start status, in competition and young horse tests, for possible use in the genetic evaluation for SWB jumping and dressage horses, respectively. Genetic parameters were estimated separately for start status in young horse tests and competitions. Also, genetic correlations of start status with lifetime accumulated points in competition and with performance traits assessed at young horse tests were estimated within discipline. Breeding values for competition performance estimated with and without inclusion of start status were compared with respect to accuracies and stallion ranking.

## 2 Material and Methods

### 2.1 Data

Pedigree information, competition records, results from the Young Horse Test (YHT) and Riding Horse Test (RHT) were obtained from the Swedish Warmblood Association. Only competition results from 2*, 3*, 4* and 5* level competitions were available (Swedish Equestrian Federation 2025). Results from lower level competitions were not included. Since 2007, all started horses in a competition have been recorded. At YHT, mainly 3‐year‐old horses are assessed for conformation, gait and jumping traits at hand or free. Four‐year‐old horses and 5‐year‐old mares that have had a foal are assessed at RHT, where the gaits are assessed under rider and jumping traits are assessed either free or under rider. In both these tests, all horses are assessed for both jumping and gait traits. However, since 2019, it is possible to choose a discipline in the RHT, provided that the horse has already participated in YHT, where all traits are assessed.

The studied population was restricted to Swedish‐born horses between 2003 and 2018 that had the possibility to compete during the period from 2007 until 2022 and therefore had a known start status in competition. Furthermore, only horses sired by a stallion with a SWB studbook number or at least 10 assessed offspring at young horse tests were included to ensure that the stallion had been active in SWB breeding. The horses had previously been categorised into four categories: jumping, dressage, allround, and Thoroughbred, depending on their sires' and grandsires' disciplines by Bonow et al. (2023). The sires' disciplines were based on their chosen participation in specialised stallion performance tests since 2002, or by pedigree, breeding values, own performance, and offspring performance. This study was restricted to jumping and dressage horses, which constituted 82% of all SWB horses born between 2003 and 2018. In total, there were 37,595 horses, of which 23,125 were jumping horses and 14,470 were dressage horses. The included horses were from 547 jumping stallions with an average of 42.3 offspring per stallion (range 1 to 888 offspring, SD 85.0), and 374 dressage stallions with an average of 38.7 offspring per stallion (range 1 to 520 offspring, SD 69.8). A pedigree file including seven ancestral generations was created and used in the genetic analyses.

### 2.2 Traits

Start status in competition was defined as started (1) or not started (0) in competition within the horse's discipline category, that is, at least one start in show jumping for jumping horses and at least one start in dressage competition for dressage horses. The horse had the possibility to start in competition from 4 years of age until the end of data collection in the year 2022. Start status in YHT, RHT, as well as start status in YHT and/or RHT (from here on called YRHT when combined) was defined as participated (1) or not participated (0) in young horse test(s).

At young horse tests, the horses are assessed on a scale from 1 (very poor) to 10 (excellent) according to the breeding objective for the traditionally evaluated traits (Viklund et al. 2008). The possibility for horse owners to choose discipline‐specific assessment in RHT if the horse has been assessed at YHT can cause missing observations for traits related to the other discipline. This was the case for less than 10% of the RHT tested horses in this study. The selection of traits for analysis in the present study was based on their discipline‐specific importance for competition performance according to previously estimated correlations (Viklund, Braam, et al. 2010). From YHT, five such traits were used in the analyses: walk at hand, trot at hand and free canter for dressage horses, and free jumping technique and temperament in free jumping for jumping horses. From RHT, six evaluated traits were used in the analyses: walk under rider, trot under rider, canter under rider and temperament under rider for dressage horses, and jumping technique and temperament in jumping for jumping horses.

Competition performance was defined as lifetime accumulated points in competition, separately for show jumping and dressage. The distribution of competition points was right‐skewed and was therefore log_10_‐transformed before the analyses (Bonow et al. 2024). Because some horses had zero points, the number 1 was added before logarithmic transformation in order to enable transformation. After transformation, skewness and kurtosis were within ±1 for both dressage and show jumping performance.

### 2.3 Statistical Methods

Data editing, descriptive statistics and phenotypic analyses were performed using SAS (Statistical Analysis System; SAS Institute Inc., 2016). Phenotypic associations between start status in YRHT and start status in competition, and between start status in YRHT and competition performance (10‐log transformed lifetime accumulated points), were investigated by comparing least squares means estimates considering sex and birth year distribution using the GLM procedure in SAS.

The fixed effects in the genetic analyses were evaluated using the GLM procedure in SAS. The analysis of variances showed that event, that is, the combination of year and location of test, was significant for all traits assessed at YHT and RHT (*p* < 0.001). Sex, defined as male or female as it was not possible to separate geldings and stallions in the data, was significant for competition traits (*p* < 0.0001), start status in competition, YHT, RHT, YRHT (*p* < 0.001) and the majority of traits assessed at YHT and RHT (*p* < 0.001 to *p* = 0.006). Age at RHT was significant for all traits (*p* < 0.001 to *p* = 0.041) except for temperament under rider during gait assessment. The effect of birth year was significant for start status in competition, YHT, RHT and YRHT (*p* < 0.001), and for competition performance traits (*p* < 0.001).

Genetic parameters were estimated using the DMU program package, version 6 (Madsen and Jensen 2013). First, a univariate threshold animal model using the Gibbs sampling algorithm was used to analyse the binary trait start status in competition within the discipline category, with the model:

(1) l=Xb+Za+e

where *I* is a vector of latent (unobserved) liabilities for start status (such that if *l*_i_ exceeds a threshold *τ*_1_ (set to 0), then *y*_i_ = 1; otherwise, *y*_i_ = 0), *X* and *Z* are incidence matrices relating the observations to fixed and random effects, respectively, vector *b* contains the fixed effects sex (male or female) and birth year of the horse (2003 to 2018), *a* is a vector of additive genetic effects of the animals and *e* is a vector of random residuals. The random effects were assumed to follow *a* ∼ *N* (0, *Aσ* ^2^_a_), where *A* is the additive genetic relationship matrix and *σ* ^2^_a_ is the additive genetic variance, and *e* ∼ *N* (0, *I*), where *I* is an identity matrix and the residual variance is fixed to 1.

Moderately vague priors (i.e., weakly informative, with degrees of freedom specified as 2) were used for the genetic variance. The Gibbs sampling algorithm was applied and run for 5,000,000 iterations, without a defined burn‐in period. Samples from every 10th iteration were drawn and passed to posterior analyses. Post‐Gibbs analysis was performed using the Gibanal 2.8 program by Van Kaam (1998) to obtain posterior statistics. The Post‐Gibbs analysis program defined a burn‐in period and performed further thinning based on serial correlations. Visual inspection of trace plots confirmed that the chains had reached convergence.

After that, linear animal models using the average information algorithm for restricted maximum likelihood were used for the further analyses. All start status traits were first analysed in univariate analyses including the same effects as in the threshold model. Genetic and phenotypic correlations of start status in competition with the young horse test traits, competition performance, and start status in YHRT were estimated in bivariate analyses within discipline category using the model.

(2) y=Xb+Za+e

where *y* is a vector with observations for the two traits (one start status trait with either competition points or young horse test scores), *X* and *Z* are incidence matrices relating the observations to fixed and random effects, respectively, vector *b* contains the fixed effects which include sex (male or female) for all traits, event (combination of year and location) for YHT (419 classes) and RHT (199 classes), age of the horse at RHT (4 or 5 years), birth year of the horse (2003 to 2018) for competition performance, *a* is a vector of additive genetic effects of the animals and *e* is a vector of random residuals. The (co)variance structures of random effects were assumed to be *a*∼*N* (0, *G*_a_ ⨂ *A*) and *e*∼*N* (0, *R*_0_ ⨂ *I*), where *A* is the additive genetic relationship matrix, *G*_a_ is the variance–covariance matrix of genetic effects, *I* is an identity matrix and *R*_0_ is the residual variance–covariance matrix.

The heritability estimates from using linear models of the all‐or‐none trait start status were transformed to the underlying continuous scale according to Dempster and Lerner (1950).

Stallions with at least 10 competing offspring within the category were ranked by breeding values for log‐10 lifetime accumulated points in show jumping or dressage estimated with and without inclusion of start status in the model. There were 212 jumping stallions with an average of 56.3 offspring competing in show jumping (range 10 to 548, SD: 71.6), and 112 dressage stallions with an average of 38.5 competing offspring in dressage competitions (range 10 to 182, SD: 38.1). Spearman's rank correlation coefficient was used to compare rankings (SAS Institute Inc. 2016). Accuracy of breeding values was defined as the correlation between true and estimated breeding value (r_TI_) and was estimated as:

$$r_{\mathrm{TI}}=\sqrt{1-\mathrm{PEV}/\sigma_{a}^{2}}$$

where *PEV* is prediction error variance.

## 3 Results

### 3.1 Phenotypic Association Between Start Status and Performance

Descriptive statistics of the data is presented in Table 1. Out of the 14,470 dressage horses, 35% had participated in YHT, 11% in RHT and 34% had started in dressage competition. Close to 6% had competed in show jumping, whereof less than half had only competed in show jumping (not shown in Table 1). The participation rate in YHT was rather constant through the years for dressage horses, while the participation in RHT declined by 53% from first to last birth year. The average age at first and last competition record in dressage was 6.3 years (SD: 2.0) and 9.7 years (SD: 3.4), respectively. Dressage horses that had participated in YRHT had a significantly (*p* < 0.001) higher participation rate in dressage competition than horses that had not participated in YHT or RHT (48% of 5718 horses vs. 25% of 8752 horses). Among the 23,123 jumping horses, 31% had participated in YHT, 10% in RHT and 56% had started in show jumping competitions. Almost 9% had competed in dressage, whereof almost three quarters had only competed in dressage. For jumping horses, there was a decrease (15%) in participation rate in YHT through the years, and the participation in RHT declined by 64% from first to last birth year. The average age at first and last competition record was 5.5 years (SD: 1.5) and 9.2 years (SD: 3.3) of age, respectively. Also for jumping horses, horses that had participated in YRHT had participated in show jumping to a larger extent: 70% of 7984 horses compared with 49% of 8752 horses (*p* < 0.001).

**TABLE 1:** Number (*N*) of included dressage or jumping Swedish Warmblood horses born between 2003 and 2018, mean, standard deviation (SD), minimum (Min) and maximum (Max) values for traits evaluated at Young Horse Test (YHT) and Riding Horse Test (RHT), competition (Comp) points in dressage or show jumping, and discipline‐specific start status^a^ in YHT, RHT, or either of the two tests (YRHT), dressage competition or in show jumping competition.

Trait	*N*	Mean	SD	Min	Max
Dressage horses (*N* = 14,470)					
YHT: walk at hand	5130	7.58	0.72	4	10
YHT: trot at hand	5130	7.60	0.80	5	10
YHT: canter, free	5130	7.47	0.77	3	10
YHT: start status in YHT	14,470	0.35	0.48	0	1
RHT: walk under rider	1584	7.27	0.76	4	9
RHT: trot under rider	1584	7.11	0.80	5	9.5
RHT: canter under rider	1584	7.25	0.85	1	10
RHT: temperament and general impression under rider	1584	7.27	0.80	4	9.5
RHT: start status in RHT	14,470	0.11	0.31	0	1
YHT or RHT: start status in YRHT	14,470	0.40	0.49	0	1
Comp: 10‐log lifetime accumulated points in dressage	4964	0.92	0.95	0	3.80
Comp: start status in dressage competition	14,470	0.34	0.47	0	1
Jumping horses (*N* = 23,125)					
YHT: jumping technique, free jumping	7101	7.50	1.08	1	10
YHT: temperament and general impression, free jumping	7101	7.41	1.25	1	10
YHT: start status in YHT	23,125	0.31	0.46	0	1
RHT: jumping technique, free jumping or under rider	2255	7.23	1.06	1	10
RHT: temperament and general impression in jumping	2255	7.29	1.15	1	10
RHT: start status in RHT	23,125	0.10	0.30	0	1
YHT or RHT: start status in YRHT	23,125	0.35	0.48	0	1
Comp: 10‐log lifetime accumulated points in show jumping	12,837	1.11	0.88	0	3.93
Comp: start status in show jumping	23,125	0.56	0.50	0	1

^a^ Start status in YHT, RHT and YRHT was defined as participated (1) or not participated (0) in young horse test(s) for horses in the dressage or jumping category separately. Start status in competition was defined as started (1) or not started (0) in competition within the horse's discipline category (i.e., for dressage horses in dressage and for jumping horses in show jumping).

Within the discipline category, horses that had competed had a significantly (*p* < 0.001) higher mean for 10‐log lifetime accumulated points in competition if they had also participated in YRHT. For dressage horses, the least squares means were 0.92 points in dressage for horses that had participated in a young horse test and 0.71 for horses that had not participated in a young horse test. The corresponding least squares means for jumping horses were 1.15 and 0.97 points in show jumping, respectively.

### 3.2 Genetic Parameters

The analyses using threshold models converged with very similar values for posterior mean, mode, and median for start status within each discipline (show jumping and dressage) (Table 2). The effective number of samples for genetic variance and heritability were 518 and 529 for dressage and 639 and 674 for show jumping, respectively. Posterior distributions of the heritability estimates for the traits were visually close to normally distributed, which was supported by distribution parameters. The mean heritability estimate from the threshold model was 0.39 for start status in dressage and 0.48 for start status in show jumping (Table 2). Using a linear model, the heritability for start status in dressage was estimated to be 0.20 on the observable 0/1‐scale and 0.34 when transformed to the underlying continuous scale. For start status in show jumping, the corresponding heritability estimates were 0.30 and 0.47. Because the heritability estimates from the threshold model were similar to the transformed heritability estimates from the linear models, linear models were subsequently used to reduce computational time. Genetic parameters from the linear models are presented in Table 3.

**TABLE 2:** Posterior mean, mode, median and standard deviation (SD) of genetic variance and heritability for start status^a^ in dressage or show jumping competition estimated with a threshold model within discipline for Swedish Warmblood dressage (*N* = 14,470) and jumping (N = 23,125) horses born between 2003 and 2018.

Trait	Genetic variance				Heritability			
	Mean	Mode	Median	SD	Mean	Mode	Median	SD
Start status in dressage	0.64	0.64	0.64	0.10	0.39	0.38	0.39	0.04
Start status in show jumping	0.95	0.90	0.94	0.13	0.48	0.48	0.48	0.03

^a^ Start status in competition was defined as started (1) or not started (0) in competition within the horse's discipline category (i.e., for dressage horses in dressage competition and for jumping horses in show jumping).

**TABLE 3:** Genetic (*σ* ^2^_a_) and residual (*σ* ^2^_e_) variances, heritability estimates on the observed scale (*h* ^2^) and transformed to underlying continuous scale (*h* ^2^_u_) for traits evaluated at Young Horse Test (YHT) and Riding Horse Test (RHT), competition (Comp) points in dressage or show jumping, discipline‐specific start status^a^ in YHT, RHT, or either of the two tests (YRHT), in dressage competition or in show jumping competition.

Trait	*σ* ^2^_a_	*σ* ^2^_e_	*h* ^2^	*h* ^2^_u_
Dressage horses (*N* = 14,470)				
YHT: walk at hand	0.22_0.02_	0.28_0__.__02_	0.44_0.04_	
YHT: trot at hand	0.36_0.03_	0.25_0.02_	0.59_0.04_	
YHT: canter, free	0.28_0.03_	0.27_0.02_	0.50_0.04_	
YHT: start status in YHT	0.07_0.01_	0.17 _< 0.01_	0.29_0.02_	0.49
RHT: walk under rider	0.15_0.04_	0.38_0.03_	0.29_0.06_	
RHT: trot under rider	0.26_0.05_	0.32_0.04_	0.44_0.07_	
RHT: canter under rider	0.24_0.05_	0.42_0.04_	0.36_0.07_	
RHT: start status in RHT	0.01 _< 0.01_	0.09 _< 0.01_	0.09_0.02_	0.15
YHT or RHT: start status in YRHT	0.08_0.01_	0.17_0.01_	0.32_0.02_	0.53
RHT: temperament and general impression under rider	0.25_0.05_	0.37_0.04_	0.40_0.07_	
Comp: 10‐log lifetime accumulated points in dressage	0.22_0.03_	0.65_0.03_	0.25_0.03_	
Comp: start status in dressage competition	0.04_0.05_	0.18_0.04_	0.20_0__.__02_	0.34
Jumping horses (*N* = 23,125)				
YHT: jumping technique, free jumping	0.28_0.03_	0.87_0.03_	0.24_0.03_	
YHT: temperament and general impression, free jumping	0.26_0.04_	1.3_0.04_	0.17_0.02_	
YHT: start status in YHT	0.09_0.01_	0.14_< 0.01_	0.41_0.02_	0.71
RHT: jumping technique, free jumping or under rider	0.54_0.08_	0.66_0__.__07_	0.45_0.06_	
RHT: temperament and general impression in jumping	0.43_0.08_	0.98_0__.__07_	0.31_0.05_	
RHT: start status in RHT	0.01 _< 0.01_	0.08 _< 0.01_	0.07_0.01_	0.11
YHT or RHT: start status in YRHT	0.10_0.01_	0.14_< 0.01_	0.42_0.02_	0.71
Comp: 10‐log lifetime accumulated points in show jumping	0.26_0.02_	0.46_0__.__02_	0.39_0.03_	
Comp: start status in show jumping	0.07_0.05_	0.17_0.004_	0.30_0.02_	0.47

*Note:* Data refer to Swedish Warmblood dressage or jumping horses born between 2003 and 2018. Parameters for start status traits were from univariate analyses and parameters for other YHT, RHT and competition traits were from bivariate analyses with start status in competition within discipline.

^a^ Start status in YHT, RHT and YRHT was defined as participated (1) or not participated (0) in young horse test(s) for horses in the dressage or jumping category separately. Start status in competition was defined as started (1) or not started (0) in competition within the horse's discipline category (i.e., for dressage horses in dressage and for jumping horses in show jumping).

Start status in YHT had higher heritability estimates than start status in RHT. For dressage horses, the estimates were 0.29 versus 0.09 on the observable scale, and 0.49 versus 0.15 when transformed to the underlying continuous scale (Table 3). For jumping horses, the corresponding estimates were 0.41 versus 0.07 on the observable scale, and 0.71 versus 0.11 when transformed to the underlying scale.

Heritability estimates for dressage‐related evaluated traits at YHT/RHT and dressage competition points, estimated in bivariate analyses with start status in dressage, varied from 0.25 (10‐log lifetime accumulated points in dressage competition) to 0.59 (YHT: trot at hand) (Table 3). Somewhat lower heritability values were estimated for jumping traits that varied from 0.17 (YHT: temperament and general impression, free jumping) to 0.45 (RHT: jumping technique, free jumping or under rider).

Genetic and phenotypic correlations between start status in dressage competition and dressage‐related traits for dressage horses, and between start status in show jumping and traits related to jumping for jumping horses, are presented in Table 4. There were strong genetic correlations between start status in show jumping and jumping traits at YHT and RHT (0.78–0.93), as well as between start status and 10‐log accumulated lifetime points in show jumping (0.98). The genetic correlations between start status in dressage and gait traits at YHT and RHT were moderate to strong (0.46–0.88), and there was a strong genetic correlation between start status and 10‐log accumulated lifetime points in dressage (0.86). Genetic correlations between start status in YRHT and start status in competition were low for show jumping (0.34) and moderate for dressage (0.67). The phenotypic correlations were all low (0.02–0.36).

**TABLE 4:** Genetic (r_g_) and phenotypic (r_p_) correlations between discipline‐specific start status^a^ in dressage or show jumping competition and evaluated traits at Young Horse Test (YHT) and Riding Horse Test (RHT), competition (Comp) points in dressage or show jumping competition, discipline‐specific start status^b^ in YHT or RHT (YRHT) for Swedish Warmblood dressage or jumping horses born between 2003 and 2018, respectively.

Trait	*r*_g_	*r*_p_
Dressage horses (*N* = 14,470)		
YHT: walk at hand	0.46_0.07_	0.12
YHT: trot at hand	0.65_0.06_	0.18
YHT: canter, free	0.71_0.06_	0.16
RHT: walk under rider	0.76_0.09_	0.19
RHT: trot under rider	0.86_0.06_	0.28
RHT: canter under rider	0.88_0.06_	0.27
RHT: temperament and general impression under rider	0.79_0.10_	0.28
YHT or RHT: start status in YRHT	0.67_0.05_	0.26
Comp: 10‐log lifetime accumulated points in dressage	0.86_0.05_	0.02
Jumping horses (*N* = 23,125)		
YHT: jumping technique, free jumping	0.85_0.04_	0.18
YHT: temperament and general impression, free jumping	0.78_0.57_	0.14
RHT: jumping technique, free jumping or under rider	0.88_0.04_	0.36
RHT: temperament and general impression in jumping	0.93_0.05_	0.35
YHT or RHT: start status in YRHT	0.34_0.04_	0.21
Comp: 10‐log lifetime accumulated points in show jumping	0.98_0.01_	0.09

^a^ Start status in competition was defined as started (1) or not started (0) in competition within the horse's discipline category (i.e., for dressage horses in dressage competition and for jumping horses in show jumping).

^b^ Start status in YHT, RHT and YRHT was defined as participated (1) or not participated (0) in young horse test(s) for horses in the dressage or jumping category separately.

### 3.3 Stallion Ranking and Breeding Value Accuracy

Stallions were re‐ranked within discipline when start status was included in the breeding value estimation for 10‐log accumulated lifetime points in dressage or show jumping. The most pronounced re‐ranking was seen for stallions with less accurate breeding values for 10‐log accumulated lifetime points in competition analysed as a single trait, in combination with either high breeding values for the competition trait and low breeding value for start status, or vice versa, from the bivariate analysis. Among the 10% highest ranked stallions, that is, 11 dressage stallions and 21 jumping stallions, eight dressage stallions and 14 jumping stallions remained in the top when start status was included in a bivariate model. The rank correlations between breeding values estimated with and without inclusion of start status were strong for both dressage stallions (0.91) and jumping stallions (0.92).

The accuracies of estimated breeding values for performance in competition increased when start status in show jumping and dressage, respectively, were included in the models. For dressage stallions, the average accuracy increased by 10%, from 0.79 to 0.88, and for jumping stallions, the average accuracy increased by 6%, from 0.88 to 0.93.

## 4 Discussion

### 4.1 Participation Rate and Preselection

The proportion of SWB jumping horses that had started in show jumping (56%) was higher than what was previously reported for both Belgian sport horses (38%) (Janssens et al. 2007) and KWPN horses (24%) (Rovere et al. 2016). The proportion of dressage horses that had started in dressage competition (34%) was also higher than in the KWPN population presented by Rovere et al. (2016) (28%) and Ducro (2010) (23%). The differences can partly depend on the definition of start status, for example, the levels of competitions included in the data. Our data were limited to competitions from 2* level and higher, meaning that horses only competing at lower levels such as local competitions were missing. An even more important reason for the difference in proportion of starting horses was likely the choice of population data used to define start status. In the present study, only jumping horses were considered for start status in show jumping, while all horses in the pedigree file were considered in the Belgian study (Janssens et al. 2007). The Belgian Warmblood horse (BWP) is known for successful horses in show jumping competitions at the high level (Chapard et al. 2023), even though equal weight is put on show jumping and dressage in the breeding goal (Koenen et al. 2004). Also, in the Dutch study by Rovere et al. (2016), the majority of the horses were used for start status in both disciplines since the registration of KWPN foals as either dressage or show jumping in the studbook was introduced in 2006, that is, at the end of the studied period. That means that a horse may never have been intended to compete in the discipline for which start status was defined, which may cause an underestimation of the frequency. Even when we divided the horses into jumping and dressage horses, some horses had been competing in other disciplines than we considered due to the rider's preference and/or the horse's talent. In other populations, such as Swedish Standardbred trotters, the horses are bred for a more uniform purpose. Furthermore, there is a larger economic incentive to start in trotting races, where owners of young trotters with approved racing performance receive an economic bonus (Svensk Travsport 2024). Also, the overall prize money is larger in racing than in equestrian competitions in Sweden.

In contrast to competition, the participation rate in YRHT was somewhat higher for dressage horses (40%) than for jumping horses (35%). These participation rates were much higher than in the study of Icelandic horses, where only 12% participated in breeding field tests (Albertsdóttir et al. 2011). Also, in comparison with other warmblood horse populations in Europe, the SWB has a high proportion of tested young horses (Thorén Hellsten et al. 2006; Chapard et al. 2023). However, we could see a decreasing participation rate in RHT throughout the years in the studied data, and for YHT, the participation rate decreased somewhat for jumping horses. If this trend continues, inclusion of the young horse test results in the genetic evaluation may contribute less to reducing selection bias for competition data (Viklund, Braam, et al. 2010), increasing the importance of handling preselection through adding a start status trait in the future.

In spite of the relatively high proportion of SWB shown in young horse tests, some preselection of horses can be assumed. It is probably the better horses that are shown at young horse tests, and this is supported by the findings that they had started in competition to a larger extent than horses not shown at young horse tests. Horses that had participated in a young horse test had on average reached significantly better competition performance results than the competing horses that had not participated in a young horse test. To include information about start status in the model when estimating breeding values for competition performance could be one way to reduce preselection bias.

### 4.2 Heritability Estimates

The heritability estimates for start status in show jumping and dressage competition were moderate on the observed scale and somewhat higher on the continuous scale, and in agreement with the study of KWPN horses by Rovere et al. (2016). The level of heritability estimates also agrees with earlier studies of Swedish, French and German trotters (Árnason 1999; Bugislaus et al. 2005; Langlois and Vrijenhoek 2004). However, Janssens et al. (2007) estimated high heritability for start status in show jumping for Belgian Warmblood horses (0.71). As mentioned above, all horses in the pedigree were used in the analyses, and the authors suspected that the high heritability could be due to the presence of dressage horses in the data. In our study, we divided the horses into dressage or show jumping horses before the analyses, which resulted in lower heritability estimates. However, with the current degree of specialisation of the SWB population, these estimates may be more accurate for the actual, largely separated, breeding populations (Bonow et al. 2023).

For start status in YRHT the heritability estimates were on a higher level compared to start status in competition. On the continuous scale, the estimates were in the same range as estimated for start status in young horse jumping contests for Belgian warmblood (Chapard et al. 2023) and in breeding field test for Icelandic horses (Albertsdóttir et al. 2011). A higher heritability compared to start status in competition could indicate that there is more environmental influence on start status in competition, such as injuries, as the horses are then older.

### 4.3 Genetic Correlations

The participation rate in show jumping was considerably higher than the participation rate in young horse tests. Different levels of preselection of competing and tested horses may also bring differences in genetic homogeneity of their genetic background. This may have contributed to the low genetic correlation between start status in YRHT and start status in show jumping competitions (0.34) for jumping horses. For dressage horses, the opposite was seen, where the participation rate of dressage horses in dressage competitions was lower than in YRHT, and the genetic correlation between start status in dressage and YRHT was moderate (0.67).

The strong genetic correlations between start status in competition and competition performance in show jumping (0.98) and dressage (0.86) showed that the competing horses were genetically better than those that did not compete. This was most obvious for show jumping. Ducro (2010) reported the same level of genetic correlation between start status and dressage for KWPN horses (0.89). However, Rovere et al. (2016) estimated lower genetic correlations for KWPN horses, but with the same trend with higher correlations for show jumping (0.82) than dressage (0.73). For trotters, genetic correlations between start status in racing and racing performance have been estimated to be between 0.32 and 0.74 (Árnason 1999; Bugislaus et al. 2005; Langlois and Vrijenhoek 2004). There may be fewer incentives to breed and keep trotters for other purposes than to start in races. The separate focus on jumping and dressage horses in the present study also likely increased the impact of talent for the specific discipline on the start status in competition, and thereby also the estimated correlations. Assuming that any systematic preselection of horses for competition is largely based on their aptitude for the sport, and that the purpose of introducing a start status trait would be to handle preselection bias, we argue that a discipline‐specific trait is of value. However, with a different aim of studying the participation rate, for example to capture effects related to the health status or general manageability of horses, a wider inclusion of horses regardless of discipline could be motivated and would likely impact the results.

For jumping horses, the high genetic correlations (0.78–0.93) between start status in show jumping and jumping traits at YHT and RHT are not unexpected as previous studies have shown that young horse test results are useful as early indicators of competition performance (Thorén Hellsten et al. 2006). For dressage horses, the correlations were at a lower level, 0.46 to 0.88, where gaits assessed under rider showed the strongest correlations with start status in dressage. This is probably because these traits are more similar to the competition situation than, for example, walk assessed at hand.

### 4.4 Ranking of Stallions and Accuracy of Breeding Values

The rank correlation of 0.92 between breeding values for competition performance, estimated with and without inclusion of start status in competition for jumping stallions, corresponds well to the results by Janssens et al. (2007) and Rovere et al. (2016). Ducro (2010) estimated a lower correlation of 0.69 between stallions' breeding values for dressage, estimated with and without inclusion of start status, whereas we found an insignificant difference between the two disciplines. In the present study, the accuracy of breeding values for dressage stallions increased more (11%) with inclusion of start status in the genetic evaluation, compared to jumping stallions (6%), this can likely be explained by the lower participation rate in dressage competitions, a stronger preselection, and consequently less accurate breeding values when preselection is not accounted for.

### 4.5 Possible Application

SWB breeding values for dressage and show jumping performance are currently based on results from all competed horses in the population and estimated in two separate multiple‐trait animal models together with relevant traits from young horse tests (Viklund et al. 2011). The inclusion of young horse test results is assumed to account to some extent for preselection to some extent of horses for competition. In the present study, we chose to define and analyse discipline‐specific start status traits to more precisely capture preselection on traits of importance for performance. Also, the number of starts and thus the proportion of horses starting in show jumping is considerably higher than in dressage competition, which could complicate analysis across disciplines in the increasingly specialised SWB population (Bonow et al. 2023). If a discipline‐specific trait for start status in competition were to be introduced in the genetic evaluation, the population has to be divided into a dressage and show jumping population. This division may be based on owners' choice or by pedigree as in Bonow et al. (2023) and in the present study.

Only SWB competition data from 2007 and onwards, when all started horses were recorded, can be taken into account in the genetic evaluation if start status would be included in the model. Viklund, Näsholm, et al. (2010) showed that accuracies of breeding values for horses born in the late period, that is, horses of interest for breeding, were unaffected when older competition data were removed from the analyses. Horses competing before 2007 would, however, receive less accurate breeding values if older data were removed, and there may be a resistance among horse owners to neglect data from older horses. However, our results indicate that the inclusion of start status may improve the genetic evaluation and benefit the selection of young, future breeding horses.

## 5 Conclusions

Discipline‐specific start status in competition and young horse tests had generally moderate to high heritability estimates in Swedish Warmblood horses. The genetic correlations between start status in competition and performance traits in both young horse tests and competition were strong for jumping horses and moderate to strong for dressage horses. Inclusion of start status in the breeding value estimation of competition performance affected stallion ranking and increased accuracies of the stallions' breeding values. Start status is a heritable trait possible to include in the genetic evaluation but may require changes before application, such as a clearer classification of horses into disciplines and removal of old data.

## Conflicts of Interest

The Swedish Warmblood Association has provided the phenotype and pedigree data for this study, and Å.G.V. has regular commitments to the Swedish Warmblood Association regarding the routine genetic evaluation. The authors declare no conflicts of interest.

## Data Availability

The data that support the findings of this study are available from Swedish Warmblood Association. Restrictions apply to the availability of these data, which were used under license for this study. Data are available from the author(s) with the permission of Swedish Warmblood Association.
